# Efficacy of psychomotor therapy for children and adolescents with anxiety disorders—a systematic literature review

**DOI:** 10.3389/frcha.2023.1182188

**Published:** 2024-01-08

**Authors:** Evelien Vriend, Janet Moeijes, Mia Scheffers

**Affiliations:** ^1^ Master Psychomotor Therapy, Windesheim University of Applied Sciences, Zwolle, Netherlands; ^2^ Department of Human Movement and Education, Windesheim University of Applied Sciences, Zwolle, Netherlands

**Keywords:** psychomotor therapy, body- and movement-based therapy, children, adolescents, anxiety disorders, systematic review

## Abstract

Specific Phobia (SP), Generalized Anxiety Disorders (GAD), and Social Anxiety Disorder (SAD) are the most prevalent anxiety disorders in children and adolescents. Although anxiety has a major influence on the body, evidence-based treatments mainly focus on cognitive and behavioral aspects of anxiety. Body- and movement-oriented interventions, such as psychomotor therapy (PMT), address the physical aspects. Bodily experience and interoceptive awareness are used to change behavior, cognition, and emotions. This review aimed to provide an overview of the efficacy of PMT for children and adolescents aged 0–18 years with SP, GAD, or SAD.

**Method:**

Data were collected in PsycINFO, Medline, Embase, ERIC, and Web of Science, from January 2020 until April 2022. Two independent researchers (EV and JM) selected the articles and performed a critical appraisal.

**Results:**

From 1,438 articles found, only one article met the inclusion criteria.

**Conclusion:**

No consensus-based statement about the efficacy of PMT in children and adolescents with SP, GAD, or SAD can be made due to the gap in the literature. Future research is needed to evaluate the efficacy. The first step may be to design treatment protocols. Subsequently, these protocols may be evaluated concerning efficacy.

## 1 Introduction

Anxiety is a normal and functional reaction of the human body to a direct or potential threat. It prepares the individual to fight or flight and consequently survive a dangerous situation (1). Likewise, anxiety is part of the healthy development of children and adolescents. Every developmental stage has its features of anxiety. For example, separation anxiety occurs in the healthy development of children aged 12–18 months, whereas anxiety for rejection is normal in adolescents aged 10–18 years. In most cases, the anxiety remains for a brief period and subsequently dissipates (2).

However, normal anxiety in childhood and adolescence may become disproportionate, and the child or adolescent may be diagnosed with an anxiety disorder. The Fifth Edition of the Diagnostic and Statistical Manual of Mental Disorders (DSM-5) describes eleven anxiety disorders in children and adolescents (1). The three most prevalent anxiety disorders in the age of 0–18 years are specific phobia (SP), generalized anxiety disorder (GAD), and social anxiety disorder (SAD) (2). These three anxiety disorders may be characterized by core features in physical, cognitive, and behavioral domains, respectively. The main difference between the disorders is the nature of anxiety and attention bias, which will be explained further.

Individuals with specific phobia experience intense and persistent fear when confronted with, exposed to, or anticipating the feared stimulus. Exposure to the feared cue results in extreme discomfort and intense bodily symptoms, reflecting the activation of the endocrine, respiratory, and autonomic nervous systems (3). Cognitive biases are displayed in individuals with SP and occur in memory, attention, expectancy, or contingency estimations (4). Dysfunctional and maladaptive beliefs cause cognitive biases about potential threats and coping abilities. This may lead to hypervigilance, and interpretation biases and will create positive feedback cycles that reinforce the maladaptive beliefs or schemas (4). The interpretation bias leads to the tendency to interpret ambiguous or neutral situations or (physical) stimuli as hostile or threatening. This results in overestimating the danger in the feared situation (1). Furthermore, the attention bias in children with an SP is away from threat-related stimuli (5). This attention avoidance of threat stimuli reinforces the tendency to perform avoidance behavior. Avoidance behavior is a common coping mechanism for fear and reducing the response to the conditioned stimuli. Therefore, avoidance behavior can be seen as maintaining a general anxiety level in individuals with SP (6).

Generalized anxiety disorders are characterized by excessive or persistent worrying. Worry is associated with enhanced neurovisceral, endocrinological, immunological, and cardiovascular activity (7). Heightened subjective levels of physiological arousal symptoms and tension are reported in individuals with GAD, indicating a possible overestimation of the basal sympathetic arousal (8). The relation between cognitive biases, interpretation biases, and hypervigilance is the same as described above for SP. However, contrary to children with SP, children with GAD point their attention towards the threat-related stimuli, resulting in an attention bias directed to the threat-related stimuli (5). Subsequently, individuals with GAD avoid their physiological arousal symptoms through excessive or persistent worrying (8).

Physical symptoms in children with social anxiety are also associated with activating the endocrine and autonomic nervous systems in reaction to the feared situation or stimuli (3). Individuals with SAD may have an adequate interoceptive accuracy but overestimate the visibility of the experienced physical symptoms and consequently associate the experienced physical symptoms with negative consequences. In addition, a biased self-perception and elevated self-focused attention cause the overestimation of social performance evaluation (9). Thereby, the attention bias is away from the threat-related stimuli (5). In reaction, individuals with SAD attempt to avoid anxious situations. This avoidance behavior can be seen as maintaining general anxiety in individuals with social anxiety (6).

SP, GAD, or SAD lead to impaired functioning in one or more domains. For instance, anxiety disorders in children and adolescents are associated with impairment in school functioning (10). Thereby, it affects cognitive, social, and behavioral development. Moreover, this is an essential risk factor for developing anxiety disorders or other mental disorders in later life, like substance use problems, subsequent anxiety, and depression (10). Therefore, adequate and timely treatment of anxiety disorders is highly relevant.

Various treatments have been developed for SP, GAD, and SAD, such as self-management tools, e-interventions, Eye Movement Desensitization and Reprocessing (EMDR), cognitive-behavioral therapy (CBT), exposure therapy, pharmacotherapy, task concentration training, and social skill training (11). However, CBT is the most used and effective treatment for anxiety disorders (12). The basic premise of CBT is that maladaptive cognitions contribute to maintaining emotional distress. Therefore, CBT aims to challenge and change maladaptive cognitions and behavioral patterns directly. First, the therapist helps a child or adolescent clarify cognitions in an anxious situation and change these maladaptive beliefs about the likelihood and actual cost of anticipated harm. Simultaneously, but of minor importance in CBT, the therapist supports the child or adolescent in recognizing anxious feelings and bodily reactions to anxiety. Second, the child or adolescent learns to cope with anxious situations and implement the skills outside the therapy setting (13).

Another commonly used treatment for anxiety disorders is exposure therapy. Exposure is also one of the techniques used in CBT protocols. However, specifically in the treatment of SP, exposure is used as a stand-alone treatment (14). Exposure is a strategy in which an individual is exposed to an anxious stimulus or situation. It aims to increase sensitivity toward physical anxiety symptoms, increase tolerance, and reduce distress (14). Exposure is efficacious for treating SP, GAD, or SAD in children and adolescents. There is a dose-response relationship between the quantity of exposure and treatment outcomes, with more time devoted to exposure linked to better results (15).

Thus, CBT uses maladaptive cognitions and associated interpretation bias as a starting point. Cognitive symptoms form the main focus, while physical and bodily components receive little attention. However, a physical starting point, with more attention to bodily symptoms of anxiety and integration of physical, cognitive, and behavioral domains of anxiety, may be beneficial in treating anxiety disorders.

Since anxiety has a significant influence on the body, focusing on the bodily effects of anxiety is relevant. Therefore, body- and movement-oriented therapies such as psychomotor therapy (PMT) focus primarily on the body. The first central element of PMT is using bodily experience as a fundament for change of behavior, cognition, and emotions. Through body- and movement-oriented interventions, new experiences will occur, and the child or adolescent will be able to adjust or practice new or alternative behavior (16). The psychomotor therapist manipulates and optimizes the context for the child or adolescent to enable these alternative and new experiences, which form the fundament for new behavior.

A second central concept of PMT is interoceptive awareness, defined as the ability of a person to interpret their bodily signals adequately (16). For instance, the psychomotor therapist arranges and manipulates a movement context through which bodily signals are stimulated. Then, with the help of the psychomotor therapist, the child or adolescent explores which bodily signals are experienced and learns to be aware of these signals. Subsequently, an individual can develop an adequate interpretation. Finally, the integration of the physical, cognitive, and behavioral domains and the integration of mind and body will be stimulated. Thereby, the interoceptive awareness of an individual is increased (17). PMT is used as adjunctive therapy for individuals of all ages with psychological or behavioral problems (16, 17, 18). PMT can be part of a multidisciplinary treatment or as a stand-alone therapy. PMT uses various movement- and body-oriented interventions to integrate them into a coherent treatment. Movement-oriented interventions are movement activities and bodily exercises, like running therapy and sports games in a social context. Body-oriented interventions are directed at bodily experiences, such as yoga, progressive muscle relaxation, and mindfulness (16).

There are several systematic reviews performed on the efficacy of PMT in adults with various mental disorders. For example, a recent systematic review and meta-analysis on body- and movement-oriented interventions in adults with posttraumatic stress disorder concluded that these interventions are effective (18). Also, a systematic review on the efficacy of body- and movement-oriented therapies targeting anger and aggression in adults with mild or borderline intellectual disabilities, concluded that body-oriented PMT is a promising approach (19). In addition, a systematic review and meta-analysis on exercise in adults with anxiety or stress-related disorders stated that exercise leads to an increase of health in general and, more specifically, a reduction of anxiety symptoms (20). Specific endurance exercise, moderate aerobic exercise, mediation and relaxation reduces anxiety disorders significantly in adults (21). Thereby, yoga can be a viable therapeutic option for reducing stage anxiety in certain situations (22).

Single experimental studies on the effect of body- and movement-oriented interventions for children and adolescents indicate that relaxation, breathing techniques and mindfulness may be beneficial for children and adolescents with an anxiety (23).

The results of the studies and systematic reviews are promising. However, there is no review of the efficacy of PMT for anxiety disorders in children and adolescents. Thus, our aim was to provide an overview of the efficacy of PMT for children and adolescents aged 0–18 years with an SP, GAD, or SAD.

## 2 Method

### 2.1 Data sources and searches

The literature was searched in the following five databases: PsycINFO, Medline, Embase, ERIC, and Web of Science, from January 2020 until April 2022. Search parameters were conducted with thesaurus. Parameters and equations are available in the Supplementary Material*.* There are no ethical implications due to the study design. There is no preregistration of the review.

Movement- and body-oriented interventions were defined as interventions that directly address patients' experiences and aim to systematically influence behaviors, cognitions, and emotions. Movement-oriented interventions are movement activities and bodily exercises, like running therapy and sports games in a social context. Body-oriented interventions are directed at bodily experiences, such as yoga, progressive muscle relaxation, and mindfulness (16).

### 2.2 Eligibility criteria

The selection of articles was based on the following inclusion criteria: (a) a quantitative and empirical study, i.e., experimental and quasi-experimental designs, such as randomized controlled trials (RCT's), controlled clinical trials (CCT's) and pretest-posttest-designs; (b) publication of the article between 1994 (publication of DSM-IV) and April 2022; (c) inclusion of at least one participant with a specific phobia, generalized anxiety disorder, or social anxiety disorder diagnosed with DSM-IV, DMS-IV-TR, or DSM-5; (d) participants aged 0–18 years; (e) primarily focus on body- or movement-oriented intervention; (f) and articles published in English or Dutch.

Articles were excluded when: (a) the diagnosis was not based on DSM-IV, DSM-IV-TR, or DSM-5; (b) anxiety as a comorbid disorder; (c) previous reviews; (d) theoretical papers; (e) and assessment-only studies. If an article examined more interventions, then only the intervention and results relevant to PMT were combined within this review. There were no restrictions for the make-up of the control group or the comparison of the effect of an intervention.

### 2.3 Study selection

Data for this study were collected using six steps. First, search parameters for keywords “specific phobia”, “generalized anxiety disorder”, “social anxiety disorder”, “body-oriented psychotherapy”, “movement-oriented psychotherapy”, and “psychomotor therapy” were conducted with thesaurus for the five databases. These keywords were merged with keywords for psychomotor therapy from an already existing file that is used in the department of Human Movement and Education. This ensures that the largest possible number of search terms could be used. Second, the search was conducted and was restricted only to yield peer-reviewed papers. Third, the search results were transferred to Rayyan to accomplish further steps. Rayyan is an online application that facilitates the screening of articles for a systematic review (24). Fourth, duplicates were removed, and the titles were censured. Fifth, yet included abstracts were read and selected. Finally, the full text was read. Steps three to five were conducted by two independent researchers. When differences in selection were found, both researchers discussed them until a consensus was reached. Discussion between both researchers was performed for a total of five studies. Reasons for discussion were the age of participants, disorder characteristics, and PMT intervention as the primary or add-on/side treatment. The discussion led to the adjustment of inclusion criteria: articles were included when PMT-intervention was the main topic, even as PMT was an add-on treatment.

The reference lists of the included articles were examined to identify additional articles to identify studies that were possibly missed. No additional studies were included.

### 2.4 Data collection

The study protocol summarized and analyzed each study regarding participant characteristics, dependent variables (i.e., type of body- and movement oriented intervention), intervention procedures and dosage, intervention outcomes, and certainty of evidence (based on tools described in 2.5 critical appraisal). The primary outcome is the anxiety symptoms of the participants. However, due to the few results, a modification to the methodology was made. Instead of displaying the relevant study parameters in a table, a descriptive technique is adopted to show the results.

### 2.5 Critical appraisal

#### 2.5.1 Methodological quality analysis

The Quality Assessment Tool for Quantitative Studies (QA) developed by the Effective Public Health Practice Project (EPHPP) was used for quality assessment (25). The QA comprises eight categories: selection bias, study design, confounders, blinding, data collection methods, withdrawals and drop-outs, intervention integrity, and analysis appropriate to the question. Various questions rank each category as strong, moderate, or weak. A global rating for the article was composed based on individual ratings. Furthermore, the QA has adequate test-retest reliability and content and construct validity (26). The ratings were performed independently by two researchers. Ratings were compared and discussed until a consensus was reached.

#### 2.5.2 Quality of evidence

The GRADE approach was used to assess the quality of evidence (27). This assessment tool rates the quality of the evidence with ratings of high, moderate, low, or very low in five different sections. Two researchers were independently involved in assessing the quality of evidence. Ratings of the GRADE approach were also compared and discussed until a consensus was made.

## 3 Results

### 3.1 Study selection

**Figure 1:**
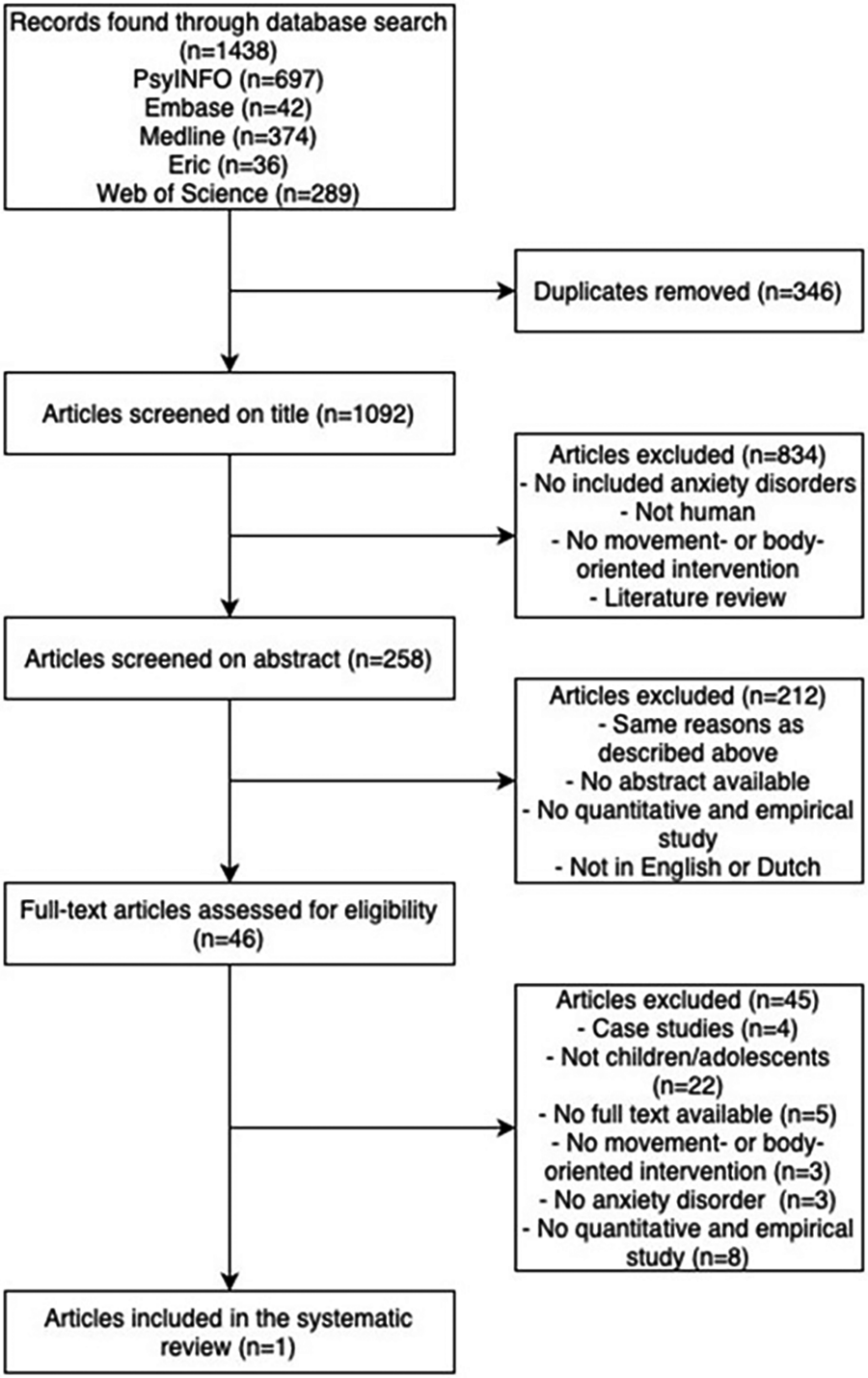
Flow diagram for the search results.

Of the 1,438 identified records, 1,092 nonduplicated were screened. After selection, one article investigating a body-oriented intervention was included in this systematic review (28). No movement-oriented interventions were found (see Figure 1).

### 3.2 General characteristics

The single included study evaluated the effect of cognitive therapy (CT) compared to relaxation therapy (RT) in children with GAD with a multiple baseline design. Participants (M = 10.7 years, *N* = 4) were assigned to the CT (*N* = 2) or RT (*N* = 2) based on their main symptoms (CT = cognitive symptoms; RT = somatic symptoms). The CT identified and modified worrisome thoughts, and the RT comprised an eleven-body-part Progressive Muscle Relaxation (PMR). Session frequency was two times a week for ten sessions. Each session consisted of an individual part (45 min), a part to mentor the parents or caregivers (30 min), and 15 min termination together. Overall, the study found a reduction of symptoms of GAD by CT and RT based on the self-report, parent report, and physiological measures. Moreover, the results show a tendency for RT to be more successful in reducing anxious arousal than CT. However, no statistical analyses were described in the article (28).

### 3.3 Quality assessment

Based on QA, the certainty of the evidence for this study is weak due to essential differences between groups prior to intervention and, therefore, the possible influence of confounders. Also, the small sample size and non-statistical statements contribute to the weak certainty of evidence.

The quality of evidence, rated by the GRADE approach, is low due to study design, risk of bias, indirectness, and imprecision. The single included study (28) incorporated some subjective outcomes. Also, no statistical statements were made, and the sample size was small. Therefore, no good comparison of the outcomes in this study can be made.

## 4 Discussion

In this systematic review we aimed to evaluate the efficacy of psychomotor therapy for children and youth aged 0–18 years with an SP, GAD, or SAD. After a rigorously and carefully executed search method, only one article met the inclusion criteria. Based on this single study, no accurate statement about the efficacy of PMT on anxiety disorders can be made. However, according to the included study, progressive muscle relaxation therapy (PMR) may effectively ease and reduce anxiety symptoms. Further research is needed to identify the efficacy of PMT for children and adolescents with an anxiety disorder.

The findings of this lone study reveal an astonishing gap in the literature. However, some relevant studies, which had to be excluded because of the inclusion criteria, were found during the research. These studies suggest positive effects of psychomotor therapy or interventions on reducing anxiety symptoms in children and (emerging) adults. For instance, one study stated that relaxation techniques—such as breathing exercises and guided relaxation—led to a significant reduction of test anxiety in children aged 8–10 years (29). Furthermore, a study on college women with social physique anxiety found a significant reduction in social physique anxiety due to yoga-group intervention (30). Thus, these results of the studies indicate a possible positive effect of psychomotor therapy for children and emerging adults in reducing anxiety disorder or anxiety symptoms. These positive effects might also be expected to emerge in children and adolescents. However, further research is necessary to confirm this hypothesis.

Our results correspond with another systematic review in which the effect of exercise and relaxation on anxiety symptoms in healthy children and adolescents were examined (31). Most of the studies included in this review were prevention studies. The authors stated that data on the effect of exercise on anxiety symptoms in children and adolescents under 16 years are sparse. The large number of prevention studies might be due to a change in the definition of health. For example, Huber et al. formulated in 2011 a new positive definition of health. They define health as “the ability to adapt and self-manage” (p.3) (32). With the new definition, attention moved from psychopathology to disease prevention and treatment to advance the individuals' resilience. This new definition might have caused a shift in research questions from treatment to prevention of mental disorders. Thus, future research is needed to identify the efficacy of PMT on anxiety disorders in children and adolescents.

A general explanation for the gap in the literature may be found in the lack of theoretical models used in the clinical field of PMT. Therapists are often guided by experience in conducting body- and movement-oriented interventions instead of theory (33). However, implementing a theoretical framework is essential in understanding and explaining the efficacy of a therapy (34). Another factor that may obstruct research is that professionals often use unclear terms or ill-defined concepts to describe the effectiveness and working mechanisms of body- and movement-oriented therapy. As a consequence, some concepts are used to describe different processes (35). It is proposed that conceptualization and operationalization are needed to contribute to developing a theory and a body of empirical evidence. Finally, few therapists use validated measures, such as questionnaires, to evaluate their therapy (33). This may be due to the limited availability of validated and reliable questionnaires. However, it obstructs the possibilities for quantitative research.

The minor use of a theoretical framework, minor conceptualization, and limited use of measurements to evaluate the efficacy of therapy are acceptable explanations for the scarcity of articles. However, the importance of further research is evident.

Finally, the last explanation for the gap in the literature is the possible influence of a publication bias. Publication bias can occur before and after submitting the article for publication and is best known for eliminating incomplete publications or negative research results (36). Therefore, the result of this review also may be caused by no publications of adverse effects.

### 4.1 Implications and future research

No clinical implications can be made, due to the gap in the literature. However, the following recommendations for future research can be made:

• Conceptualization and operationalization aiming at developing a theory and a body of empirical evidence.
• Quantitative research for creating valid and reliable questionnaires or observations to measure the efficacy of PMT in children and adolescents with SP, GAD or SAD.
• Experimental studies, such as randomized-controlled trials, on the efficacy of body- and movement-oriented interventions on children and adolescents with anxiety disorders.

## 5 Conclusion

The results of this systematic review indicate a gap in the literature. Based on our result, no consensus-based statement about the efficacy of psychomotor therapy for children and adolescents with SP, GAD, or SAD can be made. Future research is needed to explore the efficacy of PMT for children and adolescents with SP, GAD, or SAD.
