# Supplementary material for: Efficacy of psychomotor therapy for children and adolescents with anxiety disorders—a systematic literature review
Source: Front Child Adolesc Psychiatry. 2024 Jan 8;2:1182188. doi: 10.3389/frcha.2023.1182188 (PMC11731784; doi:10.3389/frcha.2023.1182188)
Supplement: Supplementary file 1 [file Datasheet1.docx]

Supplementary Material

Efficacy of Psychomotor Therapy for Children and Adolescents with Anxiety Disorders – A Systematic Literature Review

Evelien Vriend^*^, Janet Moeijes, Mia Scheffers

*** Correspondence:** Corresponding Author: e.vriend@outlook.com

# Supplementary Data

Supplement 1. Keywords PsycINFO

S1 Search terms formulated with thesaurus

((((MM "Social Anxiety") OR (MM "Social Phobia")) OR (MM "Generalized Anxiety Disorder")) OR (MM "Phobias" OR MM "Acrophobia" OR MM "Agoraphobia" OR MM "Claustrophobia" OR MM "Ophidiophobia" OR MM "School Phobia" OR MM "Social Phobia" OR MM "Acrophobia"))

S2 Search terms formulated with thesaurus, without operators DE and MM, and with operators for title, abstract and subject

TI (((("Social Anxiety") OR ("Social Phobia")) OR ("Generalized Anxiety Disorder")) OR ("Phobias" OR "Acrophobia" OR "Agoraphobia" OR "Claustrophobia" OR "Ophidiophobia" OR "School Phobia" OR "Social Phobia" OR "Acrophobia")) OR AB (((("Social Anxiety") OR ("Social Phobia")) OR ("Generalized Anxiety Disorder")) OR ("Phobias" OR "Acrophobia" OR "Agoraphobia" OR "Claustrophobia" OR "Ophidiophobia" OR "School Phobia" OR "Social Phobia" OR "Acrophobia")) OR SU(((("Social Anxiety") OR ("Social Phobia")) OR ("Generalized Anxiety Disorder")) OR ("Phobias" OR "Acrophobia" OR "Agoraphobia" OR "Claustrophobia" OR "Ophidiophobia" OR "School Phobia" OR "Social Phobia" OR "Acrophobia"))

S3 Free search terms with operators for title, abstract and subject

TI (“Social anxiety disorder” OR “SAD” OR “Specific phobia” OR “Generalised Anxiety Disorder” OR “GAD”) OR AB (“Social anxiety disorder” OR “SAD” OR “Specific phobia” OR “Generalised Anxiety Disorder” OR “GAD”) OR SU (“Social anxiety disorder” OR “SAD” OR “Specific phobia” OR “Generalised Anxiety Disorder” OR “GAD”)

S4 All combined – thesaurus and free search

S1 OR S2 OR S3

S5 Search terms formulated based on review Kruse and thesaurus

(MM "Phobias" OR MM "Acrophobia" OR MM "Agoraphobia" OR MM "Claustrophobia" OR MM "Ophidiophobia" OR MM "School Phobia" OR MM "Social Phobia")

S6 Search terms formulated based on review Kruse and thesaurus, without operators DE and MM, and with operators for title, abstract and subject

TI ("Phobias" OR "Acrophobia" OR "Agoraphobia" OR "Claustrophobia" OR "Ophidiophobia" OR "School Phobia" OR "Social Phobia") OR AB ("Phobias" OR "Acrophobia" OR "Agoraphobia" OR "Claustrophobia" OR "Ophidiophobia" OR "School Phobia" OR "Social Phobia") OR SU ("Phobias" OR "Acrophobia" OR "Agoraphobia" OR "Claustrophobia" OR "Ophidiophobia" OR "School Phobia" OR "Social Phobia")

S7 Free search terms formulated based on review Kruse with operators for title, abstract and subject

TI (“phobia” OR “phobic” OR “phobics”)

S8 All combined - Kruse

S5 OR S6 OR S7

S9 All combined – complete combination of keywords for anxiety disorders

S4 OR S8

S10 All PsyqInfo search terms for PMT

DE "Martial Arts" OR DE "Judo" OR TI ((body N1 oriented N1 therap*) OR (body-oriented N1 therap*) OR (body N1 psychotherapy*) OR (body N1 therap*) OR (breathing N1 therap*) OR (dance N1 movement N1 therap*) OR (exercise N1 therap*) OR (experiential N1 therap*) OR (movement N1 oriented N1 psychotherapy*) OR (movement N1 therap*) OR (movement-oriented N1 psychotherapy*) OR (non-verbal N1 therap*) OR (psychomotor N1 therap*) OR (psychomotric*) OR (sensorimotor N1 therap*) OR (sport N1 therap*) OR (sportstherap*) OR (Sports N1 psychiatry) OR (mind W1 body) OR (meditation N1 intervention) OR (meditation N1 approach) OR (running N1 therap*) OR yoga OR (touch N1 (therapy OR intervention)) OR (martial N1 art*) OR kickbox* OR BUDO OR AIKIDO OR HAPKIDO OR IAIDO OR “JIU-jitsu” OR JUDO OR KARATE OR KENDO OR KYUDO OR “NAGINATA-do” OR NINJUTSU OR SUMO OR “TAE kwon do” OR taekwondo OR ((Muscle OR progressive OR physical OR body OR (bio W0 feedback) OR biofeedback OR physiologic* OR neurophysiologic*) N1 relax*) OR (Relaxation N1 (Training OR intervention* OR therap*))) OR AB ((body N1 oriented N1 therap*) OR (body-oriented N1 therap*) OR (body N1 psychotherapy*) OR (body N1 therap*) OR (breathing N1 therap*) OR (dance N1 movement N1 therap*) OR (exercise N1 therap*) OR (experiential N1 therap*) OR (movement N1 oriented N1 psychotherapy*) OR (movement N1 therap*) OR (movement-oriented N1 psychotherapy*) OR (non-verbal N1 therap*) OR (psychomotor N1 therap*) OR (psychomotric*) OR (sensorimotor N1 therap*) OR (sport N1 therap*) OR (sportstherap*) OR (Sports N1 psychiatry) OR (mind W1 body) OR (meditation N1 intervention) OR (meditation N1 approach) OR (running N1 therap*) OR yoga OR (touch N1 (therapy OR intervention)) OR (martial N1 art*) OR kickbox* OR BUDO OR AIKIDO OR HAPKIDO OR IAIDO OR “JIU-jitsu” OR JUDO OR KARATE OR KENDO OR KYUDO OR “NAGINATA-do” OR NINJUTSU OR SUMO OR “TAE kwon do” OR taekwondo OR ((Muscle OR progressive OR physical OR body OR (bio W0 feedback) OR biofeedback OR physiologic* OR neurophysiologic*) N1 relax*) OR (Relaxation N1 (Training OR intervention* OR therap*))) OR SU ((body N1 oriented N1 therap*) OR (body-oriented N1 therap*) OR (body N1 psychotherapy*) OR (body N1 therap*) OR (breathing N1 therap*) OR (dance N1 movement N1 therap*) OR (exercise N1 therap*) OR (experiential N1 therap*) OR (movement N1 oriented N1 psychotherapy*) OR (movement N1 therap*) OR (movement-oriented N1 psychotherapy*) OR (non-verbal N1 therap*) OR (psychomotor N1 therap*) OR (psychomotric*) OR (sensorimotor N1 therap*) OR (sport N1 therap*) OR (sportstherap*) OR (Sports N1 psychiatry) OR (mind W1 body) OR (meditation N1 intervention) OR (meditation N1 approach) OR (running N1 therap*) OR yoga OR (touch N1 (therapy OR intervention)) OR (martial N1 art*) OR kickbox* OR BUDO OR AIKIDO OR HAPKIDO OR IAIDO OR “JIU-jitsu” OR JUDO OR KARATE OR KENDO OR KYUDO OR “NAGINATA-do” OR NINJUTSU OR SUMO OR “TAE kwon do” OR taekwondo OR ((Muscle OR progressive OR physical OR body OR (bio W0 feedback) OR biofeedback OR physiologic* OR neurophysiologic*) N1 relax*) OR (Relaxation N1 (Training OR intervention* OR therap*)))

S11 Combined – complete search

S9 AND S10

Supplement 2. Keywords Medline

S1 Search terms formulated with thesaurus

"social anxiety disorder" OR (MM "Phobia, Social") OR (MM "Phobic Disorders+") OR "social anxiety" OR "SAD" OR "Generalized anxiety disorder" OR "generalised anxiety disorder" OR "GAD" OR "specific phobia" OR "specific anxiety"

S2 Search terms formulated with thesaurus, without operators DE and MM, and with operators for title, abstract and subject

TI ("social anxiety disorder" OR ("Phobia, Social") OR ("Phobic Disorders+") OR "social anxiety" OR "SAD" OR "Generalized anxiety disorder" OR "generalised anxiety disorder" OR "GAD" OR "specific phobia" OR "specific anxiety") OR AB ("social anxiety disorder" OR ("Phobia, Social") OR ("Phobic Disorders+") OR "social anxiety" OR "SAD" OR "Generalized anxiety disorder" OR "generalised anxiety disorder" OR "GAD" OR "specific phobia" OR "specific anxiety") OR SU ("social anxiety disorder" OR ("Phobia, Social") OR ("Phobic Disorders+") OR "social anxiety" OR "SAD" OR "Generalized anxiety disorder" OR "generalised anxiety disorder" OR "GAD" OR "specific phobia" OR "specific anxiety")

Remark: no free search terms were formulated. All search terms with operators for title, abstract and subject are included in the thesaurus terms. Thereby, the review of Kruse did not add new search terms. Everything was included by Phobic Disorders +

S3 Free search terms formulated based on review Kruse with operators for title, abstract and subject

TI ("phobia" OR "phobias" OR "phobic" OR "phobics") OR AB ("phobia" OR "phobias" OR "phobic" OR "phobics") OR SU ("phobia" OR "phobias" OR "phobic" OR "phobics")

S4 All combined – complete combination of keywords for anxiety disorders

S1 OR S2 OR S3

S5 All Medline search terms for PMT

MH "Martial Arts" OR TI ((body N1 oriented N1 therap*) OR (body-oriented N1 therap*) OR (body N1 psychotherapy*) OR (body N1 therap*) OR (breathing N1 therap*) OR (dance N1 movement N1 therap*) OR (exercise N1 therap*) OR (experiential N1 therap*) OR (movement N1 oriented N1 psychotherapy*) OR (movement N1 therap*) OR (movement-oriented N1 psychotherapy*) OR (non-verbal N1 therap*) OR (psychomotor N1 therap*) OR (psychomotric*) OR (sensorimotor N1 therap*) OR (sport N1 therap*) OR (sportstherap*) OR (Sports N1 psychiatry) OR (mind W1 body) OR (meditation N1 intervention) OR (meditation N1 approach) OR (running N1 therap*) OR yoga OR (touch N1 (therapy OR intervention)) OR (martial N1 art*) OR kickbox* OR BUDO OR AIKIDO OR HAPKIDO OR IAIDO OR “JIU-jitsu” OR JUDO OR KARATE OR KENDO OR KYUDO OR “NAGINATA-do” OR NINJUTSU OR SUMO OR “TAE kwon do” OR taekwondo OR ((Muscle OR progressive OR physical OR body OR (bio W0 feedback) OR biofeedback OR physiologic* OR neurophysiologic*) N1 relax*) OR (Relaxation N1 (Training OR intervention* OR therap*))) OR AB ((body N1 oriented N1 therap*) OR (body-oriented N1 therap*) OR (body N1 psychotherapy*) OR (body N1 therap*) OR (breathing N1 therap*) OR (dance N1 movement N1 therap*) OR (exercise N1 therap*) OR (experiential N1 therap*) OR (movement N1 oriented N1 psychotherapy*) OR (movement N1 therap*) OR (movement-oriented N1 psychotherapy*) OR (non-verbal N1 therap*) OR (psychomotor N1 therap*) OR (psychomotric*) OR (sensorimotor N1 therap*) OR (sport N1 therap*) OR (sportstherap*) OR (Sports N1 psychiatry) OR (mind W1 body) OR (meditation N1 intervention) OR (meditation N1 approach) OR (running N1 therap*) OR yoga OR (touch N1 (therapy OR intervention)) OR (martial N1 art*) OR kickbox* OR BUDO OR AIKIDO OR HAPKIDO OR IAIDO OR “JIU-jitsu” OR JUDO OR KARATE OR KENDO OR KYUDO OR “NAGINATA-do” OR NINJUTSU OR SUMO OR “TAE kwon do” OR taekwondo OR ((Muscle OR progressive OR physical OR body OR (bio W0 feedback) OR biofeedback OR physiologic* OR neurophysiologic*) N1 relax*) OR (Relaxation N1 (Training OR intervention* OR therap*))) OR SU ((body N1 oriented N1 therap*) OR (body-oriented N1 therap*) OR (body N1 psychotherapy*) OR (body N1 therap*) OR (breathing N1 therap*) OR (dance N1 movement N1 therap*) OR (exercise N1 therap*) OR (experiential N1 therap*) OR (movement N1 oriented N1 psychotherapy*) OR (movement N1 therap*) OR (movement-oriented N1 psychotherapy*) OR (non-verbal N1 therap*) OR (psychomotor N1 therap*) OR (psychomotric*) OR (sensorimotor N1 therap*) OR (sport N1 therap*) OR (sportstherap*) OR (Sports N1 psychiatry) OR (mind W1 body) OR (meditation N1 intervention) OR (meditation N1 approach) OR (running N1 therap*) OR yoga OR (touch N1 (therapy OR intervention)) OR (martial N1 art*) OR kickbox* OR BUDO OR AIKIDO OR HAPKIDO OR IAIDO OR “JIU-jitsu” OR JUDO OR KARATE OR KENDO OR KYUDO OR “NAGINATA-do” OR NINJUTSU OR SUMO OR “TAE kwon do” OR taekwondo OR ((Muscle OR progressive OR physical OR body OR (bio W0 feedback) OR biofeedback OR physiologic* OR neurophysiologic*) N1 relax*) OR (Relaxation N1 (Training OR intervention* OR therap*)))

S6 Combined – complete search

S4 AND S5

Supplement 3. Keywords Embase

S1 Search terms formulated with thesaurus

'social phobia'/exp/mj OR 'phobia'/exp/mj OR 'social anxiety'/exp/mj OR 'generalized anxiety disorder'/exp/mj OR 'generalised anxiety disorder 7'/exp/mj OR 'specific phobia'/exp/mj

S2 Search terms formulated with thesaurus, without operators DE and MM, and with operators for title, abstract and subject

social phobia:ti OR phobia:ti OR social anxiety:ti OR generalized anxiety disorder:ti OR generalised anxiety disorder 7:ti OR specific phobia:ti OR social phobia:ab OR phobia:ab OR social anxiety:ab OR generalized anxiety disorder:ab OR generalised anxiety disorder 7:ab OR specific phobia:ab OR social phobia:kw OR phobia:kw OR social anxiety:kw OR generalized anxiety disorder:kw OR generalised anxiety disorder 7:kw OR specific phobia:kw

S3 Free search terms with operators for title, abstract and subject

social anxiety disorder:ti OR SAD:ti OR GAD:ti OR specific anxiety:ti OR social anxiety disorder:ab OR SAD:ab OR GAD:ab OR specific anxiety:ab OR social anxiety disorder:kw OR SAD:kw OR GAD:kw OR specific anxiety:kw

S4 All combined – thesaurus and free search

S1 OR S2 OR S3

S5 Search terms formulated based on review Kruse and thesaurus

'phobia'/exp/mj

S6 Search terms formulated based on review Kruse and thesaurus, without operators DE and MM, and with operators for title, abstract and subject

phobia:ti OR phobia:ab OR phobia:kw

S7 Free search terms formulated based on review Kruse with operators for title, abstract and subject

phobias:ti OR phobic:ti OR phobics:ti OR phobias:ab OR phobic:ab OR phobics:ab OR phobias:kw OR phobic:kw OR phobics:kw

S8 All combined - Kruse

S5 OR S6 OR S7

S9 All combined – complete combination of keywords for anxiety disorders

S4 OR S8

S10 All Embase search terms for PMT

combat sport:kw or boxing:kw or kickboxing:kw or exp martial art:kw OR ((body ADJ1 oriented ADJ1 therap*) OR (body-oriented ADJ1 therap*) OR (body ADJ1 psychotherapy*) OR (body ADJ1 therap*) OR (breathing ADJ1 therap*) OR (dance ADJ1 movement ADJ1 therap*) OR (exercise ADJ1 therap*) OR (experiential ADJ1 therap*) OR (movement ADJ1 oriented ADJ1 psychotherapy*) OR (movement ADJ1 therap*) OR (movement-oriented ADJ1 psychotherapy*) OR (non-verbal ADJ1 therap*) OR (psychomotor ADJ1 therap*) OR (psychomotric*) OR (sensorimotor ADJ1 therap*) OR (sport ADJ1 therap*) OR (sportstherap*) OR (Sports ADJ1 psychiatry) OR (mind W1 body) OR (meditation ADJ1 intervention) OR (meditation ADJ1 approach) OR (running ADJ1 therap*) OR yoga OR (touch ADJ1 (therapy OR intervention)) OR (martial ADJ1 art*) OR kickbox* OR BUDO OR AIKIDO OR HAPKIDO OR IAIDO OR “JIU-jitsu” OR JUDO OR KARATE OR KENDO OR KYUDO OR “NAGINATA-do” OR NINJUTSU OR SUMO OR “TAE kwon do” OR taekwondo OR ((Muscle OR progressive OR physical OR body OR (“bio feedback”) OR biofeedback OR physiologic* OR neurophysiologic*) ADJ1 relax*) OR (Relaxation ADJ1 (Training OR intervention* OR therap*))):ti:ab:kw

S11 Combined – complete search

S9 AND S10

Supplement 4. Keywords Eric

S1 Search terms formulated with thesaurus

DE "School Phobia"

S2 Search terms formulated with thesaurus, without operators DE and MM, and with operators for title, abstract and subject

TI (“School phobia”) OR AB (“School phobia”) OR SU (“School phobia”)

S3 Free search terms with operators for title, abstract and subject

TI (“Social anxiety” OR “Social anxiety disorder” OR “Social phobia” OR “SAD” OR

“Generalised anxiety disorder” OR “Generalized anxiety disorder” OR “GAD”

OR “Specific phobia” OR “Specific anxiety”)OR AB (“Social anxiety” OR “Social anxiety disorder” OR “Social phobia” OR “SAD” OR “Generalised anxiety disorder” OR “Generalized anxiety disorder” OR “GAD”OR “Specific phobia” OR “Specific anxiety”) OR SU (“Social anxiety” OR “Social anxiety disorder” OR “Social phobia” OR “SAD” OR “Generalised anxiety disorder” OR “Generalized anxiety disorder” OR “GAD” OR “Specific phobia” OR “Specific anxiety”)

S4 Free search terms formulated based on review Kruse with operators for title, abstract and subject

TI (“phobic” OR “phobics” OR “phobia” OR “phobias”) OR AB (“phobic” OR “phobics” OR “phobia” OR “phobias”) OR SU (“phobic” OR “phobics” OR “phobia” OR “phobias”)

Remark: the review of Kruse did not add new search terms formulated with thesaurus.

S5 All combined – complete combination of keywords for anxiety disorders

S1 OR S2 OR S3 OR S4

S6 All Eric search terms for PMT

TI ((body N1 oriented N1 therap*) OR (body-oriented N1 therap*) OR (body N1 psychotherapy*) OR (body N1 therap*) OR (breathing N1 therap*) OR (dance N1 movement N1 therap*) OR (exercise N1 therap*) OR (experiential N1 therap*) OR (movement N1 oriented N1 psychotherapy*) OR (movement N1 therap*) OR (movement-oriented N1 psychotherapy*) OR (non-verbal N1 therap*) OR (psychomotor N1 therap*) OR (psychomotric*) OR (sensorimotor N1 therap*) OR (sport N1 therap*) OR (sportstherap*) OR (Sports N1 psychiatry) OR (mind W1 body) OR (meditation N1 intervention) OR (meditation N1 approach) OR (running N1 therap*) OR yoga OR (touch N1 (therapy OR intervention)) OR (martial N1 art*) OR kickbox* OR BUDO OR AIKIDO OR HAPKIDO OR IAIDO OR “JIU-jitsu” OR JUDO OR KARATE OR KENDO OR KYUDO OR “NAGINATA-do” OR NINJUTSU OR SUMO OR “TAE kwon do” OR taekwondo OR ((Muscle OR progressive OR physical OR body OR (bio W0 feedback) OR biofeedback OR physiologic* OR neurophysiologic*) N1 relax*) OR (Relaxation N1 (Training OR intervention* OR therap*))) OR AB ((body N1 oriented N1 therap*) OR (body-oriented N1 therap*) OR (body N1 psychotherapy*) OR (body N1 therap*) OR (breathing N1 therap*) OR (dance N1 movement N1 therap*) OR (exercise N1 therap*) OR (experiential N1 therap*) OR (movement N1 oriented N1 psychotherapy*) OR (movement N1 therap*) OR (movement-oriented N1 psychotherapy*) OR (non-verbal N1 therap*) OR (psychomotor N1 therap*) OR (psychomotric*) OR (sensorimotor N1 therap*) OR (sport N1 therap*) OR (sportstherap*) OR (Sports N1 psychiatry) OR (mind W1 body) OR (meditation N1 intervention) OR (meditation N1 approach) OR (running N1 therap*) OR yoga OR (touch N1 (therapy OR intervention)) OR (martial N1 art*) OR kickbox* OR BUDO OR AIKIDO OR HAPKIDO OR IAIDO OR “JIU-jitsu” OR JUDO OR KARATE OR KENDO OR KYUDO OR “NAGINATA-do” OR NINJUTSU OR SUMO OR “TAE kwon do” OR taekwondo OR ((Muscle OR progressive OR physical OR body OR (bio W0 feedback) OR biofeedback OR physiologic* OR neurophysiologic*) N1 relax*) OR (Relaxation N1 (Training OR intervention* OR therap*))) OR SU ((body N1 oriented N1 therap*) OR (body-oriented N1 therap*) OR (body N1 psychotherapy*) OR (body N1 therap*) OR (breathing N1 therap*) OR (dance N1 movement N1 therap*) OR (exercise N1 therap*) OR (experiential N1 therap*) OR (movement N1 oriented N1 psychotherapy*) OR (movement N1 therap*) OR (movement-oriented N1 psychotherapy*) OR (non-verbal N1 therap*) OR (psychomotor N1 therap*) OR (psychomotric*) OR (sensorimotor N1 therap*) OR (sport N1 therap*) OR (sportstherap*) OR (Sports N1 psychiatry) OR (mind W1 body) OR (meditation N1 intervention) OR (meditation N1 approach) OR (running N1 therap*) OR yoga OR (touch N1 (therapy OR intervention)) OR (martial N1 art*) OR kickbox* OR BUDO OR AIKIDO OR HAPKIDO OR IAIDO OR “JIU-jitsu” OR JUDO OR KARATE OR KENDO OR KYUDO OR “NAGINATA-do” OR NINJUTSU OR SUMO OR “TAE kwon do” OR taekwondo OR ((Muscle OR progressive OR physical OR body OR (bio W0 feedback) OR biofeedback OR physiologic* OR neurophysiologic*) N1 relax*) OR (Relaxation N1 (Training OR intervention* OR therap*)))

S7 Combined – complete search

S5 AND S6

Supplement 5. Keywords Web of Science

S1 Free search terms with operators for title, abstract and subject su

TI=(“Social phobia” OR “Social anxiety” OR “Social anxiety disorder” OR “SAD” OR “Generalised anxiety disorder” OR “Generalized anxiety disorder” OR “GAD” OR “specific phobia” OR “specific anxiety”) OR TS=(“Social phobia” OR “Social anxiety” OR “Social anxiety disorder” OR “SAD” OR “Generalised anxiety disorder” OR “Generalized anxiety disorder” OR “GAD” OR “specific phobia” OR “specific anxiety”)

S2 Free search terms formulated based on review Kruse with operators for title, abstract and subject

TI=(“phobia” OR “phobias” OR “phobic” OR “phobics”) OR TS=(“phobia” OR “phobias” OR “phobic” OR “phobics”)

S3 All combined – complete combination of keywords for anxiety disorders

S1 OR S2

S4 All Web of Science search terms for PMT

TS=((body NEAR/1 oriented NEAR/1 therap*) OR (body-oriented NEAR/1 therap*) OR (body NEAR/1 psychotherapy*) OR (body NEAR/1 therap*) OR (breathing NEAR/1 therap*) OR (dance NEAR/1 movement NEAR/1 therap*) OR (exercise NEAR/1 therap*) OR (experiential NEAR/1 therap*) OR (movement NEAR/1 oriented NEAR/1 psychotherapy*) OR (movement NEAR/1 therap*) OR (movement-oriented NEAR/1 psychotherapy*) OR (non-verbal NEAR/1 therap*) OR (psychomotor NEAR/1 therap*) OR (psychomotric*) OR (sensorimotor NEAR/1 therap*) OR (sport NEAR/1 therap*) OR (sportstherap*) OR (Sports NEAR/1 psychiatry) OR (mind NEAR/1 body) OR (meditation NEAR/1 intervention) OR (meditation NEAR/1 approach) OR (running NEAR/1 therap*) OR yoga OR (touch NEAR/1 (therapy OR intervention)) OR (martial NEAR/1 art*) OR kickbox* OR BUDO OR AIKIDO OR HAPKIDO OR IAIDO OR “JIU-jitsu” OR JUDO OR KARATE OR KENDO OR KYUDO OR “NAGINATA-do” OR NINJUTSU OR SUMO OR “TAE kwon do” OR taekwondo OR ((Muscle OR progressive OR physical OR body OR (“bio feedback”) OR biofeedback OR physiologic* OR neurophysiologic*) NEAR/1 relax*) OR (Relaxation NEAR/1 (Training OR intervention* OR therap*)))

S5 Combined – complete search

S3 AND S4
